# Social inequalities, psychosocial influences, and symptom development in patients with irritable bowel syndrome - a longitudinal qualitative analysis of the SOMA.SOC study

**DOI:** 10.1186/s12889-025-25628-2

**Published:** 2025-11-26

**Authors:** Rieke Barbek, Anna Christin Makowski, Olaf von dem Knesebeck

**Affiliations:** Institute of Medical Sociology, University Medical Center Hamburg-Eppendorf, Hamburg, Germany

**Keywords:** Influence and impact, Irritable bowel syndrome, Psychosocial factors, Qualitative longitudinal research, Social inequalities, Symptom development

## Abstract

**Background:**

Despite the individual and economic relevance of irritable bowel syndrome (IBS), research on social inequalities in the development of IBS symptoms is lacking. Therefore, the aim of the study was to shed light on (1) IBS symptom development across different social identities, and (2) psychosocial influences on and impact of IBS symptom development to understand the processes by and the social contexts in which these experiences are created.

**Methods:**

Analyses made use of a prospective longitudinal qualitative study with three serial time points (initial interview, three-months, and twelve-month follow-up). Interviews of *n* = 20 participants were included capturing a variety of social identities (occupational status, gender, history of migration), symptom specifics, and experiences with and in relation to their IBS symptomatology. Following a longitudinal qualitative research (LQR) approach, deductive and optional inductive themes were extracted.

**Results:**

Over the one-year study period, participants’ symptoms largely improved, undulated, remained mainly stagnant, or worsened. Social inequalities became apparent in that participants with a high occupational status and males experienced improvements in their symptoms compared to participants with a low occupational status and females, respectively. To understand these differences, the following deductive themes related to psychosocial influences on and impact of IBS symptom development were identified: stressors regarding private environment (e.g., life events, familial responsibilities) and livelihood, coping, and perceived stigma in medical contexts.

**Conclusions:**

To mitigate social inequalities in IBS symptom development, both, individuals’ coping strategies and social power need to be strengthened, especially for those with intersecting social identities associated with oppression, like low socioeconomic status, female gender, or a history of migration. Thereby, work-related, medical, and psychoeducational interventions are needed addressing both the individual and structural level.

**Supplementary Information:**

The online version contains supplementary material available at 10.1186/s12889-025-25628-2.

## Background

Irritable Bowel Syndrome (IBS) is a prevalent functional gastrointestinal disorder characterized by recurrent abdominal pain or discomfort associated with altered bowel habits, such as diarrhea, constipation or a mixed type of both [1]. Globally, IBS prevalence estimates vary between 4 and 10% [2], and its impact extends beyond physical symptoms, affecting individuals in their quality of life, relationships, social activities, job fulfillment, and financial security [3, 4]. Also on societal level, IBS imposes a considerable economic burden on healthcare systems and society [5, 6].

The development of IBS symptoms is often chronic and characterized by relapses, with considerable inter-individual variability in the type and severity of symptoms [7]. IBS symptoms commonly follow a cyclical pattern, where flare-ups of abdominal pain, bloating, and changes in bowel habits may alternate with periods of remission [8]. Studies suggest that the diagnosis of IBS is relatively stable after initial evaluation, but a small proportion of patients (2–5%) may be diagnosed with alternative organic gastrointestinal (GI) disorders over time [9]. In observational studies, longer follow-up periods showed that IBS worsened in 2–18% of patients, while about a third reported the entire disappearance of symptoms over a median period of two years, and the remaining patients experienced a stagnation of symptoms [9]. A study by Mearin et al. examined the clinical patterns of IBS patients over a three-month period and also highlighted the instability of symptoms and differences in severity, displaying substantial intra-individual variability [10].

IBS is recognized as a disorder of gut-brain functions, with complex and multifactorial aetiology. This perspective acknowledges the interplay between visceral hypersensitivity, alterations in the communication pathways between the enteric and central nervous system, and the profound influence of psychosocial factors [11, 12]. This shift in understanding underlines the need to explore not only the biological underpinnings of IBS but also the intricate ways in which psychosocial factors contribute to its development, experience, and management. In the biopsychosocial model, psychosocial factors, such as (occupational) stress, anxiety, depression, social support, and stigma, are understood to influence digestive function, the perception of symptoms, illness behavior, and ultimately, health outcomes for individuals with IBS [13–15].

Social inequalities in health refer to existing but avoidable differences in health status and access to healthcare resources between different population groups [16]. Following an intersectional approach, these include individuals with various interwoven, often oppressed social identities, including gender, race/history of migration, and socioeconomic status (SES), among others [17, 18]. SES, which is typically assessed using indicators such as income, education, and occupation, is recognized as a fundamental determinant of health. Individuals with lower SES typically experience poorer health, reduced well-being, and increased rates of illness and death compared to those with higher SES [16]. Also, in the context of IBS, understanding social inequalities is relevant. Regarding SES and its indicators, so far results are mixed. One study indicates higher IBS prevalence among individuals with higher levels of education [19]. Other research reports associations between IBS and indicators of SES, such as low income, as well as low educational attainment [20, 21]. Additionally, there is evidence that individuals with lower SES face increased exposure to various psychosocial factors, which have also been found to be associated with onset and development of IBS symptoms. According to these studies, there are associations between IBS and (early) childhood trauma, the experience of stressful life events, psychological distress, lack of social support, low health literacy, unemployment, and occupational stressors or job strain [13, 15, 22]. Further social inequalities in IBS become apparent regarding gender-related differences, as studies reveal that women are affected by IBS twice as often as men [2]. Moreover, compared to men, women with IBS seem to experience more intense symptoms and report higher levels of psychological distress as well as shortcomings in diagnosis and treatment [23, 24]. In this context, it should also be noted that men have been found more reluctant to seek medical care compared to females, which can influence health outcomes and reporting [25]. Next to socioeconomic and gender aspects, also comorbidities such as functional somatic syndromes (e.g. fibromyalgia) [8] and cultural factors play a role in diagnosis and treatment of IBS. The latter have so far not been well described [26]. Data relating to IBS based on ethnicity indicated variations in the diagnosed rates and symptom development among different racial and ethnic groups. Some research suggests that Black and Hispanic individuals may have lower diagnosed rates compared to white individuals [27]. Differences in symptom patterns have also been observed across ethnic groups [28]. In this context, differences in healthcare provision and management of IBS should be noted. In the United States, for example, specific ethnic groups, including Black, Hispanic, and Asian patients, have been reported to be less likely to receive consultations with dietitians or gastroenterologists and more likely to undergo gastroenterology procedures compared to white patients [29]. The authors interpret this not as a sign of better access to care, but as an indication of healthcare disparities rooted in impaired communication between minority patients and their predominantly white medical providers and structural discrimination.

It is reasonable to theorize that social inequalities play a role in shaping IBS symptom development, which is known to be sensitive to stress and psychosocial influences, among others. Investigating this connection is crucial for a comprehensive understanding of the burden of IBS and for developing not only interventions that timely address the needs of all affected individuals but also broader preventive approaches. The present study utilizes longitudinal qualitative research (LQR), which is indispensable for providing in-depth insights into subjective symptom development. The main phenomenon of interest in LQR are lived experiences of *time and change.* Understanding the processes by which these experiences are created is a key strength of LQR. Thereby, LQR sheds light on multiple *influences* on these processes, *impact* of change, and the *context* in which these processes emerge [30].

Against this background, the present study addresses the following research questions:

1. How do individuals with different social identities (occupational status, gender, and history of migration) experience their IBS symptoms over time? Does this reveal social inequalities?
2. Are there differences in psychosocial influences on and impact of IBS symptom development among patients with various social identities?

## Methods

### Study design

This study is part of a project on social inequalities in aggravating factors of persistent somatic symptoms (SOMA.SOC), carried out within the Research Unit 5211 “Persistent SOMAtic Symptoms ACROSS Diseases: From Risk Factors to Modification (SOMACROSS)” [31, 32]. The study design incorporates both cross-sectional quantitative and longitudinal qualitative data. The methodical details of the vignette-based cross-sectional population survey were published in the study protocol [32]. The qualitative part of the study covered longitudinal interviews at three measurement points (initial interview, six- and twelve-months follow-up), with participants affected by IBS. To examine social inequalities, participants were stratified by gender (male/female), occupational status (high/low), and history of migration (yes/no). Occupational status was operationalized using the International Socio-Economic Index of Occupational Status (ISEI) [33], based on the International Standard Classification of Occupations (ISCO-08) [34]. Scores were dichotomized to organize participants into high and low occupational status. History of migration was defined in accordance with the German Federal Statistical Office, including individuals who immigrated themselves or have at least one parent who immigrated [35].

Participant recruitment employed a multi-stage, purposive sampling strategy. Firstly, patients with IBS were identified from a general medical outpatient clinic registry and contacted by a collaborating physician. Following informed consent, eligible patients were contacted by the research team for further screening (please see below). The collaborating physician received an expense allowance for the enrolled participants. In addition, an invitation to participate in the study was posted on the SOMACROSS homepage and a cooperation with another SOMACROSS sub-project focusing on gastrointestinal complaints like IBS [31] was launched. Lastly, several (medical) institutions in Hamburg and surrounding areas were contacted via telephone and/or email to inform about the study. Relevant institutions included both general practitioners and polyclinics, either involved in the medical care of patients with IBS or located in neighborhoods with a relevant proportion of people with a history of migration. These institutions were listed in a doctor search engine of the Hamburg association of statutory health insurance physicians. Also, advisory centers with a focus on the subject of migration were contacted. Participating institutions distributed study leaflets to inform about the study and people interested could then contact the research team via telephone or email. After providing further information on the telephone and checking for eligibility (please see below), eligible participants received a study information package and consent form via mail. Participants received a €20 expense allowance per interview. Compensation was provided on a per-interview basis, as approved by our ethics committee. This ensured participants were fairly compensated for their participation, regardless of whether they completed all follow-up interviews.

Inclusion criteria were: an ICD-10 diagnosis of IBS by a medical doctor, age ≥ 18 years, German language proficiency, and provision of written informed consent. Exclusion criteria included severe medical conditions requiring immediate intervention, florid psychosis, substance abuse disorders, and acute suicidality. In addition, to realize a balanced sample, people were considered non-eligible when they did not meet the stratification criteria (gender, occupational status, and history of migration).

### Data collection

Participant enrollment and carrying out the interviews lasted from January 2023 to September 2024. The targeted sample size of *n* = 24 (*n* = 3 per stratum consisting of gender x occupational status x history of migration) was initially overestimated [32]. Data collection persisted until a minimum of two initial interviews per stratum were achieved. An exception had to be made within the stratum of males with a high occupational status and a history of migration, where, despite extended recruitment efforts, the target sample size could not be reached. Nonetheless, the final sample size of *n* = 21 participants at T0 exceeded the minimum sample size of twelve interviews recommended for qualitative research [36]. At six-month follow-up (T1) sample size was *n* = 20 and at twelve-month follow-up (T2) *n* = 19, resulting in k = 60 interviews. Despite greatest effort, loss to follow-up was due to the fact that the participants could not be reached. The participant lost at T1 had to be excluded, whereas data from the participant lost at T2 could still be included in the analysis. During the recruitment phase, *n* = 31 persons expressing their interest were considered ineligible due to various reasons, including failure to meet diagnostic criteria (71.0%), language proficiency (9.7%), and non-fulfillment of stratification criteria (9.7%). Furthermore, *n* = 2 persons refused to participate or could not be reached due to outdated contact data.

Semi-structured telephone interviews were held following an interview guide consisting of open-ended questions (see Table 1). Follow-up questions were asked to explore emerging topics in greater depth. The precise phrasing and sequence of questions was adaptable, allowing for the comprehensive exploration of main topics while maintaining flexibility to address individual-specific issues. To integrate issues of time into follow-ups, the interview guide was supplemented by questions about development since the last interview. Field notes were taken ad hoc, capturing particularly important observations, reflections on the overall impression of the participant, and relevant points for future interviews. After each interview, researchers were given time for reflection. In preparation for the follow-up interviews, the interviewer retrieved the previous interview(s) for each participant. Participants were then invited to reflect on specific aspects from the previous interviews to enhance participant’s reflexivity regarding time and change. Prior to implementation, the interview guides underwent pre-testing with both a volunteer patient who was not included in the study and members of the research team. This process facilitated the identification and rectification of redundancies and the refinement of question specifications.

**Table 1:** Topics of the interview guide

Topics	Specification
1) Origin, causes and development of the symptoms	Symptom perception, severity of symptoms, health anxiety *+ development over time (T1 and T2)*
2) Coping and help-seeking	Behavior and experiences in dealing with symptoms, health literacy, treatment experiences, treatment expectations *+ development over time (T1 and T2)*
3) Social interaction and perception by others	Disclosure of symptoms, public stigma and perceived discrimination associated with the symptoms *+ development over time (T1 and T2)*

With participants’ verbal consent, interviews were audio-recorded. Interviews were transcribed using noScribe, an open-source artificial intelligence-based audio transcription software [37]. The automatically generated transcripts were edited by two project team members against pre-established uniform transcription rules using the transcription software f4 [38]. Interviews were set to last about 30 min; overall duration ranged from 9 to 56 min, with a median duration of 24 min and the tendency towards shorter interviews at follow-up.

### Data analysis

After all interviews had been completed and transcribed, they were analyzed using LQR according to Saldaña [30]. As mentioned above, LQR sheds light on the processes of *change through time* and on multiple *influences* on these processes, *impact* of change, and the *context* in which these processes emerge. In particular, Saldaña proposes sixteen questions related to three subcategories: framing questions regarding context and conditions that influence changes, descriptive questions characterizing types of changes, and interpretive questions portraying the behavior of interest within its context of relationships. To utilize the studies’ research questions, following Saldaña, *change* was defined as IBS symptom development over time. For each participant, IBS symptoms were extracted at each of the interviews and compared intra-individually to determine the development of symptoms. Thereby, symptoms could improve, undulate, stagnate, or worsen. The symptom development was then determined as follows: ‘largely improved’ (including improved, stagnant, and/or undulating symptoms); ‘undulating’ (including improved, undulating, stagnant, and/or worsened symptoms); ‘mostly stagnant’ (including stagnant and/or undulating symptoms); ‘worsened’ (including undulating, stagnant, and/or worsened symptoms). The participants’ social identities made up of occupational status, gender, and history of migration constituted the *context*. We conducted a hybrid analysis, primarily using a deductive, or top-down, approach based on the explanatory framework for the biopsychosocial etiology of IBS by to Van Oudenhove and collegues [15]. This framework provided our core themes for psychosocial *influences* (sources, intensifiers, and mitigators) and psychosocial *impact*. These were in line with the working model of the SOMACROSS research unit, which investigates risk factors and mechanisms for somatic symptom persistence [31]. While our analysis was predominantly deductive to ensure consistency with existing theory, we also maintained an inductive, or bottom-up, lens. This allowed us to remain open to and identify any new themes emerging from the data that were not captured by the initial framework. Our longitudinal qualitative approach is most closely aligned with a reflexive thematic analysis, as it enabled us to identify evolving themes and meanings while participants’ experiences changed over time. The phenomenon of change was analyzed twofold: first, over time, comparing differences in IBS symptoms between time points for each participant to determine symptom development. Second, through time, following a topic across time, participants, and strata of social identities (particularly occupational status) to understand the context, influences, and impact of change.

An iterative process of text work, categorization, coding, analysis, and presentation of findings was employed in accordance with Saldaña’s LQR approach and further concretized with the qualitative content analysis by Kuckartz [39]. To enhance intersubjective reliability, a consensual coding process was implemented by two independent coders [39]. Initially, a coding system was developed based on a subset of transcripts, and code segments were independently assigned. Subsequently, the coders engaged in a collaborative process to resolve discrepancies, ensuring consistency and precision. This collaborative phase continued until inter-coder agreement was reached for all interviews. The finalized coding system comprised deductive and inductive categories, each with a sub code to assess change over time. This coding system was finally applied to all transcripts by the two researchers, including subsequent comparisons and consensus discussions as needed. In addition, memo writing, specifically coding rule memos, code memos, and participant summaries across all three points in time played a crucial role in the analytical process.

Illustrative quotes from participants are presented in the results section, with each quote accompanied by a key consisting of participant’s number; participant’s social identities; symptom development; points in time of interview: #PX; F (female), M (male), hO (high occupational status), lO (low occupational status), nMh (no history of migration), Mh (history of migration); symptom development: i (largely improved), u (undulating), s (mainly stagnant), w (worsened); initial interview (T0), six-month follow-up (T1), twelve-month follow-up (T2).

The iterative analysis process was performed with the software MAXQDA 24 [40], which allowed for ongoing analytical reflection throughout the research process.

## Results

### Study sample

A purposively selected sample of *n* = 20 participants, representing diverse social identities (as shown in Table 2), was analyzed in this study. Half of the sample consisted of females, was aged 40 + years, and had a high occupational status. The vast majority of participants worked either part- or full-time.

Concerning their diagnosis, 55% of the sample stated that they had been given a diagnosis of IBS in the past five years. The onset of symptoms ranged from more than 10 years ago (45%) to less than 5 years ago (25%).

**Table 2:** Participant characteristics (*n* = 20; T0)

	*n*	%
Gender		
Female	11	55.0
Male	9	45.0
Age		
< 40	10	50.0
40+	10	50.0
Range	22–69	
History of migration		
No	10	50.0
Yes	10	50.0
Occupational status		
High	10	50.0
Low	10	50.0
Employment		
Yes (full/part-time)	18	80.0
No (including seeking work, retired)	2	20.0
Onset of symptoms		
< 5 years	5	25.0
5–10 years	6	30.0
> 10 years	9	45.0
Date of diagnosis		
< 5 years	11	55.0
5–10 years	6	30.0
> 10 years	2	10.0
n.s.	1	5.0

*n.s.* not specified

The type and intensity of the experienced symptoms varied both inter- and intra-individually. At T0, the majority of patients experienced stool irregularities such as diarrhea, although some were also affected by constipation or experienced a mixed form. In addition, participants described recurring pain, such as abdominal cramps or a general feeling of discomfort, bloating, and flatulence.

Some participants also described other complaints that they saw in the context of their IBS. These included exhaustion, heartburn, nausea, and loss of appetite. In addition, some of the individuals were affected by comorbidities. These included, for example, food intolerances, endometriosis, atopic eczema, or mast cell activation syndrome – but also cancer, headache and migraine, or adhesions in the abdominal region due to prior surgeries.

### IBS symptom development

Among the participants, symptoms developed quite differently over time (see Table 3). In many cases, symptoms improved, albeit at different points in time and to varying degrees. Often, symptoms already began to improve before the first interview (preT0). *“End of treatment [RB: gut hypnosis] coincided with my retirement and as a result the situation improved very slowly but at least it improved.” (#P26_MhOnMh_i_T0).*

**Table 3:** IBS symptom development (*n* = 20; preT0-T2)

Socio-demographics			Participant number #	preT0				T0	T1	T2	Symptom development (preT0-T2)
Occupational status	Gender	History of migration		1985–1999	2000–2009	2010–2019	2020–2023				
high	male	2nd	P8			xD		+	~	+	largely improved
	female	1st	P19			xD		+	~	+	largely improved
	female	nMh	P7				xD	+	~	=	largely improved
	female	1st	P10			xD		+	~	=	largely improved
	male	nMh	P13			xD		+	~	=	largely improved
	male	nMh	P26			x	D	+	~	=	largely improved
	male	nMh	P4		xD			+	~	n.s.	largely improved
	female	nMh	P15				xD	+	=	~	largely improved
	male	nMh	P16			x	D	~	~	+	largely improved
	female	1st	P11			xD		+	-	~	undulating
low	male	2nd	P18				xD	+	+	~	largely improved
	male	2nd	P17			x	D	+	~	~	largely improved
	female	nMh	P12				xD	~	=	+	largely improved
	female	nMh	P1	x		D		-	+	~	largely improved
	female	2nd	P3	x		D		+	+	-	undulating
	female	1st	P2		x		D	+	=	-	undulating
	female	1st	P14			xD		+	~	-	undulating
	male	nMh	P30				xD	=	=	~	mostly stagnant
	female	nMh	P6		x		D	=	=	-	worsened
	male	2nd	P5			xD		-	-	~	worsened

Points in time: before initial interview (preT0), initial interview (T0), six-month follow-up (T1), twelve-month follow-up (T2). Symptoms + improved, ~ undulated, = stagnated, - worsened

*x* onset of symptoms, *D* time of diagnosis, *nMh* no history of migration, 1 st immigrated themselves, 2nd parent(s) immigrated, *n.s*. not specified

In contrast, in some cases, namely participants #P1, #P12 and #P16, symptoms started to improve during follow-ups. Although the symptoms improved overall, (short) phases of undulation due to various reasons like stress or diet, among others, were common. This explains the phrasing ‘largely improved’.

*“Sometimes a little softer*,* when you’re stressed*,* that’s clear. The bowel is a nervous system*,* but for the most part*,* let’s say 80% of the time*,* I have solid stools*,* which I haven’t known for years. I am of the opinion that this is due to the adhesions.” (#P1_FlOnMh_i_T2)*.

For other participants, however, symptoms initially improved, but then worsened substantially. Participants’ descriptions illustrate a kind of undulating symptom development with alternating phases of improvement and worsening. *“It [antihistamine] helped me for a while*,* but then the symptoms came back.” (#P3_FlOMh_u_T2)* In one case, there was no relevant change in symptoms throughout follow-ups, picturing stagnation of symptoms. *“There was once a phase where I had the impression that it had become a little less or a little more solid. But that was only for a short time and then it was actually back to the old state.” (#P30_MlOnMh_s_T2)* In another case, the initially stagnant symptoms considerably worsened by the final follow-up.

*“I got rid of this heartburn*,* but now I have other complaints*,* I’m constantly nauseous. And what I also have to deal with extremely is that I constantly have diarrhea*,* so bad in fact that I often have to turn back when I’m on my way to work. And that’s why I had another colonoscopy a fortnight ago and was diagnosed with the fact that my intestine is too long. And now I always have to take psyllium*,* which doesn’t help at all.” (#P6_FlOnMh_w_T2)*.

Another participant’s symptoms markedly worsened during the first two interviews. By the end of follow-up, the symptoms stabilized at a low level, though they temporarily worsened during a Covid-19 infection. The latter two cases are exemplary of worsened symptom development during the study period.

Noteworthy, participants with a high occupational status showed a largely improved trend in symptoms compared to those with a low occupational status. Among the latter, symptoms more often undulated, stagnated, or worsened over time. In addition, several participants with a low occupational status reported quite long time gaps between the onset of symptoms and the diagnosis. Besides, males seemed to be better off regarding symptom development compared to females, as their symptoms more often improved over time. Conversely, undulating symptom development was only observed in females who have a history of migration. Regarding participants’ explanations of symptom development, it is striking that participants with a low occupational status often lacked explanations, both regarding psychosocial as well as biomedical aspects. In contrast, all of the participants with a high occupational status recognized potential influences on symptom development over the course of time.

### Psychosocial influences of and impact on IBS symptom development

Throughout the interview process, five deductive themes that related to psychosocial influences of and impact on IBS symptom development were identified. Patterns in relation to occupational status emerged for these themes (detailed in Table 4).

**Table 4:** Psychosocial influences of and impact on IBS symptom development (*n* = 20; preT0-T2)

Psychosocial influences and impact	Occupational status	Symptom development largely improved	undulating	mainly stagnant	worsened
Stressors – private environment					
	high	#P4 caring for young children (Int; preT0), pressure to perform for children (T1)//#P7 big argument with partner (S; preT0), expectations of others (Int; T1), breakup (M; T2)//#P13 pressure to perform for child, managing family (Int; T0-T2)//#P16 caring for/death of frail parents (Int; preT0), dinner invitations (Imp; T0-T1)//#P19 taking care of family in warzone, sick parents (S, Int; preT0-T2)//#P26 difficulties going out, on vacation (Imp; preT0-T2)	#P11 going out to meet new people (Imp; preT0), reducing hobbies with partner, e.g. camping, cooking (Imp; preT0-T2), organization of marriage (Int, Imp; T1), family planning with endometriosis (Imp; T2)		
	low	#P1 death of partner as only social contact (Int; preT0), overburdened by organizational issues, e.g. related to estate administration (Int; preT0-T2); lack of supportive contacts (Imp; preT0-T2)//#P12 overdo social activities (Int; T2)	#P3 reducing dinner invitations, spontaneous activities (Imp; preT0-T2)		#P5 stressful partnership (S; preT0), reducing social contacts, reducing leaving apartment to a minimum (Imp; T1-T2), family planning impossible (Imp; T0)
Stressors – livelihood					
	high				
	low	#P1 financial worries due to reduced working hours (Int, Imp; preT0-T2)//#P17 financial worries due to unemployment (Imp; preT0-T2) and possible disability pension (Imp; T2)	#P14 existential worries due to job insecurity (Int; preT0-T1) and insolvency (Int; T2), finding a job feasible to symptoms (Imp; T2)		#P5 existential worries due to reduced working hours (Imp; T0), sick leave (Imp; T1), unemployment (Imp; T2), finding a job feasible to symptoms (Imp; T2)
Coping – improving working conditions					
	high	#P4 career move (M; T1)//#P7 career planning after doctorate (Int; T1-T2)//#P8 reducing working hours (M; T1)//#P13 working remote instead of distressing business trips (M; preT0)//#P15 home office (preT0-T2)	#P11 change of distressing job (Int, M; T1)		
	low				
Coping – understanding and managing symptoms and own personality					
	high	#P7 acceptance stress association, finding own rules (M; preT0-T2)//#P8 finding own rules (M; preT0)//#P10 acceptance stress association, finding own rules (M; preT0-T2)//#P19 acceptance stress association, finding own rules (M; preT0-T2)			
	low	#P12 acceptance stress association, finding own “rules” (M; preT0-T2)	#P2 acceptance, only regular check-ups (preT0)		
Perceived stigma in medical contexts (intersectional underlined)					
	high	#P13 receiving no treatment after diagnosis, being labeled (preT0)//*#P15* being told to be too young for colonoscopy, being belittled, not taken seriously (preT0)//#P16 being belittled, not taken seriously (preT0-T2)	*#P11* experiencing unwillingness to help, instead being referred to other medical specialties, being psychologized, not taken seriously, no appointments, experiencing omission of diagnostics due to foreign surname & female gender (preT0-T2), being advised on pregnancy as solution (T1-T2)		
	low	#P1 being psychologized, not taken seriously, experiencing lack of sympathy (preT0-T2)//#P12 being psychologized, not taken seriously, experiencing lack of diagnostic and support, unhelpful advice (preT0)//#P17 being psychologized (preT0-T2)	*#P2* too young for colonoscopy, not taken seriously, being dismissed, experiencing lack of understanding (preT0)//#P3 being psychologized, receiving unhelpful advice (preT0-T2)	#P30 having been given information only upon own request (T2)	#P5 being psychologized (preT0)//#P6 being belittled, being withheld operation, being fobbed off with painkillers (preT0-T2)

Participant number: #PX. Participant’s perception regarding influence on/impact of symptoms: S source, Int intensifier, M mitigator, Imp impact; others not specified. Points in time: before initial interview (preT0), initial interview (T0), six-month follow-up (T1), twelve-month follow-up (T2)

### Stressors - private environment

In the thematic domain of stressors regarding the private environment, participants articulated both daily hassles and life events concerning familial responsibilities (e.g., caring for young children or frail parents, the death of a partner), the lack of commitment regarding hobbies and activities, and the reduction of social interaction. These were mentioned by various participants whose symptoms developed quite differently, both occupational statuses, and at different points in time. However, participants with a high occupational status and largely improved symptoms more often reported that these stressors, particularly taking on responsibility for others, intensified their symptoms or triggered flare-ups.

*“Then there was the personal situation with the war*,* because I’m originally from Ukraine. I really had a lot to deal with last year*,* with my relatives*,* things I couldn’t control. And then plus my job. And then at the end of the year I was really exhausted. Not just with my stomach*,* but also with other things in general. With migraines. I was totally irritated and exhausted. And now I’ve taken three months off*,* where I’ve slept a lot. So I really withdrew from life. As far as I could*,* of course. Because the private situation didn’t allow that 100%. Because I have two parents who are both over eighty. My mum is very ill. So that’s the part where I can’t switch off. But I gave some of it to my sister. So as far as I could with the private situation*,* I pulled myself out of it. And I also stepped back completely professionally. Almost completely. About 90%. I’m feeling better now. […] It’s stressful*,* but I’m trying to balance it out.” (#P19_FhOMh_i_T0)*.

In contrast, participants whose symptoms did not improve (which in some cases corresponds to a low occupational status) more often reported symptoms affecting their social life at different points in time, e.g., social exclusion or a lack of social support.

*“I’ve also had difficulties with social contacts all my life because I was ill and I was invited to a wedding or something or somewhere for dinner and I was always ill afterwards and then I always said I couldn’t go or I couldn’t come because I couldn’t eat the food*,* and then people withdrew from me.” (#P1_FlOnMh_i_T0)*.

Besides, a stressful partnership was mentioned as a source for the beginning of symptoms. *“I’ve only had it since I got this boyfriend. […] Because we had a big fight one weekend and that’s when it started.” (#P7_FhOnMh_i_T0).* In other cases, however, the influence on symptoms remained unclear. *“I’ve often asked myself what the relation is*,* because when I’m on holiday and I’m not stressed and everything*,* then I have it*,* too.” (#P3_FlOMh_u_T2)*.

### Stressors – livelihood

Participants with a low occupational status invariably referred to stressors regarding livelihood as impact of their symptoms – including financial concerns due to job insecurity, sick leave or unemployment. Across the different participants, a social and financial decline became apparent over time. This began with repeated, sometimes prolonged sick leave and/or reduced working hours due to symptoms. As time and sick leave progressed, job loss may have occurred. Finding a new job compatible with individual symptoms and personal restrictions was described as challenging. As a result, unemployment persisted, sometimes with the prospect of an early disability pension.

*“I used to work full-time*,* but that’s no longer possible. I can only work part-time now. And then you may have to call in sick on certain days or for a longer period of time. Then I may also have to change work areas because it’s simply no longer feasible to work in the same area as before. And that makes a big difference. Financially*,* when you go from full-time to part-time. And you don’t necessarily make yourself popular with your employer. He’s not really keen either.” (#P5_MlOhM_w_T0)*.

*“But now I have to make sure that since I am chronically ill*,* I can find a suitable job with this illness. That makes it very difficult to find a job. I have a restriction for now*,* I can no longer do every job. I can’t do my old job either. Now I have to look for a job that suits me. Plus*,* I now have the problem that there are gaps in my CV. You can tell that they’re due to illness or unemployment.” (#P5_MlOMh_w_T2)*.

During follow-up interviews, one participant repeatedly described this occupational insecurity as an intensifier of symptoms.

*“Influenced by my current situation*,* so stress. Well*,* not stress*,* more like: what’s happening*,* where should I go? You speculate a lot of things in your head. Should I give up everything now and retire*,* I could take early retirement in October. And then it’s a burden*,* it’s not enough money*,* then you get deductions and so on. You have a lot on your mind. There is no peace of mind. And then it turns up*,* the pain in the stomach or in the abdomen.” (#P14_FlOMh_u_T2)*.

### Coping – improving working conditions

On the contrary, participants with a high occupational status reported different ways of improving their working conditions as a coping strategy, which positively influenced their IBS symptoms over time. Reducing working hours, for example, alleviated stress without triggering existential worries. Likewise, as participant #P19 indicated above, taking several months off was beneficial. Also, the transition to working remote reduced stress associated with travelling.

*“I would say that stress is still a bit of a problem*,* but I’ve counteracted that a bit with a 4-day week and a bit more balance and I think that*,* in combination with the change in diet*,* this makes me less susceptible to stress when you’ve slowed down a bit.” (#P8_MhOMh_i_T1)*.

Also, career advancement was seen in relation to a generally higher level of life satisfaction accompanied by an improvement in symptoms.

*“I want to be there for the children*,* function and not be restricted in any way. I have a positive effect. I’m starting a new job next week and I’m really looking forward to it. I’ve been with a company for eight years and now I’m taking a small career step. That may also go hand in hand with my current*,* improved well-being. That there is a general positive development in my life right now.” (#P4_MhOnMh_i_T1)*.

Furthermore, finding a new, less stressful job was initially stressful for one participant and temporarily worsened the symptoms of that person. However, it proved manageable and reduced work-related stress in the long term.

### Coping – understanding and managing symptoms

Another important coping strategy referred to the participants’ understanding and managing of symptoms and also of their own personality. Throughout the study, participants with a high occupational status particularly seemed to recognize and accept an association between their symptoms and (acute or prolonged) stress. This was described as a process which evolved over a longer period of time, with the onset already before initial interviews. This process was accompanied by the necessity and participants’ capability to find their own rules to manage their symptoms. These rules included particularly, but not exclusively, managing stress and diet.

*“Because*,* as with the other illness*,* I have learnt that as long as I stick to certain rules and am sensitive to my current stress level*,* for example in relation to my work. I think I can actually adapt most things for myself through my diet. I used to go to the seaside with my friends at the weekend*,* sit in the sun and drink one bottle of wine after the next*,* to put it bluntly. Just what you do for a weekend. And since I’ve had IBS*,* my alcohol consumption has plummeted. Because I just know I can’t do it. So I’m not doing myself any favors. That’s okay*,* too. So that’s what I mean*,* I now understand the rules of the game. And I know what I can do. And what I and my body are good at. And I’ll push myself to the limit and try to stay within this framework. That’s why I’m actually fine with it.” (#P7_FhOnMh_i_T0)*.

For some participants, this also included recognizing and accepting their own anxious personality and/or striving for control. By the final interviews, participants seemed to demonstrate adherence with their rules since these helped to better deal with (meanwhile mitigated) symptoms.

*“I have now started taking part in a parent-child swimming class with our son. It started two or three weeks ago. It was just that I was very excited before the first appointment*,* because I usually*,* I think I’ve also said before*,* that especially in situations where I don’t know what to expect in detail*,* I have a certain amount of tension. Which then also leads to a bit of stress and slight symptoms appear*,* so that I realize*,* oh*,* now my stomach is rumbling a bit. I try to eat foods that are easy to digest*,* maybe a ripe banana*,* so that I’m not completely on an empty stomach. And then it’s usually like this*,* I know that if I have to endure the next 20 or 30 minutes until we’ve arrived at the swimming pool and changed*,* I have to ‘endure it’ and then it’s fine again. And as soon as we were there*,* it was all over. (#P13__MhOnMh_i_T2)*

Thereby, during follow-ups, the temporary worsening of symptoms – sometimes in the form of extreme flare-ups – necessitated some participants to put together and follow their own rules even more strictly.

*“And I realized that I was reaching my limits so that I was becoming ill. And that’s also the point where it got me thinking. Well*,* radical. I don’t want to say that I hadn’t thought about it before. But now it really was a very radical situation where I said*,* I have to focus on myself*,* not on others. I can’t save the world.” (#P19_FhOMh_i_T2)*.

In another case, however, acceptance was described more in terms of accepting the symptoms as undulating and beyond one’s control.

*“There was a time when I was very worried. I thought I had cancer. Or something even worse. But all the studies were good. And they didn’t find anything. So I’m reassured now*,* I would say. I don’t know if that’s such a good thing. Because somehow you accept the symptoms. And say*,* ok*,* that’s just the way it is. But*,* yes*,* I worried a lot.” (#P2_FlOMh_u_T0)*.

### Perceived stigma in medical contexts

Several participants, specifically those whose symptoms did not improve, referred to perceived stigma in medical contexts. This included experiences of labeling and psychologization, condescending communication, unhelpful advice and/or prescriptions, and the withholding of further diagnostics and/or medical care, among others.

*“But I went to the doctor about it and he told me to google the internet. And then I thought ‘yes*,* that’s great advice*,* that’s why I went to see you.’ I’ve also never heard of a doctor telling you to go online because of your symptoms.” (P6_FlOnMh_T1).*

Particularly participants with a low occupational status reported being affected by psychologization.

*“You don’t get any further. You notice that with GPs. When they say you’ve come because you have an uneasy stomach or a stomach ache*,* which is actually a bit of a stupid word. Well*,* then they give you Lefax [RB: remedy for bloating*], *then they come with whatever. And do you have stress then? No*,* I’m not stressed*,* I’m not really stressed. Yes*,* try to be a bit calmer. And then that’s it. So I don’t think you’re really taken seriously. Especially with abdominal pain*,* it’s always a bit like that*,* it always goes straight to the psyche*,* just like irritable bowel syndrome. You can get into this psychological number relatively quickly.” (#P3_FlOMh_u_T2)*.

In addition, only female participants perceived intersectional stigma due to their gender, (young) age and/or history of migration.

*“I was also alone in the emergency ward a few times*,* it took a long time*,* I was sent back home with Ibu [RB: painkiller]. Then I was with my boyfriend and it went really quickly*,* I was taken seriously*,* the doctors also talked to him. Yes*,* it’s just different. So*,* somehow*,* when he’s there*,* I’m taken seriously. And if not*,* then not. […] To be honest*,* I’m going to change my name now and I hope that when I have a German name it will be a bit better. I also have a doctorate*,* I will also have that on my ID*,* maybe I will be treated a bit better if I have a doctorate and a German name.” (#P11_FhOMh_u_T1)*.

Participants also explained that they had refrained from seeking medical care for their IBS symptoms, partly because of the perceived stigmatization and the fact that the consultations did not help to improve their symptoms.

*“I’ve tried so many things in the past and*,* as I said*,* so many doctors appointments and I don’t feel like it has really helped. And now I’m a bit more in the mode of okay*,* it’s no use anyway and just causes stress and I’m constantly ignored anyway or I’m constantly told you should just get pregnant and then everything will be fine. I really don’t feel like having anything like that anymore. I’ve cried enough. […] I went to the GP once when the stomach pains were really bad. We had a bit of a look*,* […] no ultrasound or anything*,* just a bit of palpation and okay. I tried it or I just checked that I wasn’t dying.” (#P11_FhOMh_u_T1)*.

Taken together, the findings from our longitudinal qualitative analysis illustrate the dynamic interplay between psychosocial factors and symptom experience in the context of social inequalities.

## Discussion

### Summary and interpretation

To the best of our knowledge, this is the first longitudinal qualitative study on social inequalities in IBS symptom development. In particular, the study investigated IBS symptom development as lived experiences of time and change. Also influences on and impact of symptom development were explored to understand the processes by and the social contexts in which the experiences are created. For this purpose, a sample of *n* = 20 participants was analyzed capturing a variety of social identities, symptom specifics, and experiences.

Over the one-year study period, participants’ IBS symptoms developed quite differently, which was subsumed in largely improved, undulating, mainly stagnant, or worsened symptoms. Social inequalities were reflected by participants with a high occupational status experiencing considerably large improvements in their symptoms in contrast to participants with a low occupational status. A similar pattern was observed for males compared to females, further indicating gender-related inequalities. In addition, intersectional inequalities became apparent, as especially females with a history of migration faced undulating symptoms. While a direct gender-based comparison was not the primary focus of our analysis, gender was considered as a key stratifying variable alongside occupational status and migration history to explore the intersectional nature of social inequalities.

The variability of symptom development found in this study is in line with other, primarily quantitative, studies, pointing out inter- and intra-individual disparities in type and severity of IBS symptoms over time [9, 10]. Regarding social inequalities, research is yet inconclusive; however, increasing evidence indicates an association between IBS and low SES [18–20] as well as female gender [2].

Following Saldaña’s LQR approach [30] and in accordance with the biopsychosocial aetiological model of IBS [15, 31], five deductive themes related to psychosocial influences on and impact of IBS symptom development were identified, in which patterns of social inequalities emerged. No further inductive themes arose. Stressors regarding the personal environment were faced by almost all participants and were somehow linked to the symptoms (as source, intensifier, mitigator, or impact). However, participants with a low occupational status seemed more affected by their symptoms throughout the study. The symptoms limited not only their social life, but also led to existential worries, social and financial decline due to prolonged sick leave, reduced working hours, or a difficult job search. Already several decades ago, such a decline was observed in the context of serious mental illnesses and described as the social drift hypothesis [41]. The association between IBS and unemployment, low education and female gender was highlighted, for instance, in the study by Farzaneh et al. [20].

In contrast, only participants with a high occupational status referred to the possibility of improving working conditions, such as a career move, working part-time or remote. This ability to consider or make such changes in turn positively influenced symptoms over time. It is well-documented that higher occupational positions typically enable greater influence over job design and working conditions due to increased autonomy, control, specialized knowledge, negotiating power, and more direct access to decision-makers. Employees in these positions often have the opportunity, the resources, and the motivation to shape their work environment to optimize their performance and enhance their job satisfaction [42, 43].

Also, understanding and managing symptoms and own (anxious) personality traits was elaborated on predominantly by participants with a high occupational status as another coping strategy. This might be explained by various reasons, such as higher health literacy including the idea and acceptance of a biopsychosocial health approach, more and well-educated social contacts/networks for discussion and support, and more time, financial resources, and social power to stand up for one’s own needs in higher SES groups [44–46].

Perceived stigma in medical contexts was experienced by several participants irrespective of social identities. For instance, participants reported condescending communication, unhelpful advice and prescriptions, and the withholding of further diagnostics or medical care. These experiences are in line with the review of Hearn et al. [14]. It is noteworthy that participants with a low occupational status seemed particularly affected by psychologization. Furthermore, female participants, partially with a history of migration, experienced intersectional stigma. Intersectional stigma was initially researched in HIV and only recently in mental illnesses [47]. In the publication on perceived discrimination in fatigue, we detected intersectional stigma [48], but there is a lack of specific research on intersectional stigma in IBS.

The reported experiences of perceived stigma were related to the participants’ decision to cease seeking medical care, but not directly with symptom development. Nonetheless, it can be assumed that the diagnostic gap and an ongoing uncertainty regarding possible explanations for symptom development are partially attributed to these stigmatizing experiences and unsatisfactory medical consultations. These issues predominantly impact participants with a low occupational status and unimproved symptoms. This is in concordance with IBS stigma research, which indicates an association between perceived stigma and worse health outcomes, quality of life, and delayed or avoided help-seeking, and also highlights gender bias [14, 49]. Further gender bias becomes evident in IBS-like diseases, e.g. inflammatory bowel disease or endometrioses, pointing to misdiagnosis in females [50, 51]. Research also suggests that healthcare providers may be more likely to interpret symptoms reported by male patients as organic and symptoms reported by female patients as psychosocial [49].

### Strengths and limitations

Despite its advantages, LQR is still not used as a standard approach in (qualitative) health research. Accordingly, this study is one of the first that examines IBS symptom development over time, looking differentially at influences and impact through the lens of social inequalities. Interviewing patients throughout the course of their illness, however, can give a much better picture of their experiences than a single interview. Although the study covered a one-year period only, in many interviews important events occurred, which the participants rated as relevant in the context of their IBS symptoms. However, this was not the case for all interviews, explaining the partly short interview duration. That said, the observation period must not be unduly long in order to obtain reliable self-reported data. It should be noted that in the context of IBS, depending on the course of symptoms, a study period of even one year may already be too long for patients to accurately recall the detailed ebb and flow of their symptoms. Moreover, LQR is specifically suitable to develop an ongoing relationship between the participant and researcher (which became apparent in low loss to follow-up), facilitating discussions about sensitive and personal issues. This trusting participant-researcher relationship further enhanced a reflexive process by placing issues of time in the interview, e.g., asking participants to reflect on the content of their previous interviews. This process was continued with the reflexivity of researchers after the interviews. Nonetheless, in a few cases, participants seemed unable to fully cope with reflective questions. Answering these in their entirety, indeed, requires a certain ability for abstraction and linguistic nuances that cannot be expected from everyone, e.g., people for whom German is not their mother tongue. The reflection constituted one important part of data triangulation. Since resources were limited, neither transcripts nor the reported main findings were returned to participants for correction or discussion.

Despite greatest recruitment efforts, it can be assumed that the study sample does not fully cover the heterogeneity of individuals affected by IBS and their lived experiences – which is not the aim of qualitative research. Further selection bias may exist, as individuals with stronger opinions and greater openness to the research topic may have been more inclined to participate. In addition, it must be noted that specifically stigmatized social identities (for example, gender minorities, refugees or those who fall outside the welfare system) were not reached by this study. Nonetheless, the study sample was quite diverse regarding occupation, overall health status, features, duration and severity of IBS symptoms. Despite the diversity and ample number of participants in this qualitative study, we would like to point out that the findings should be interpreted with caution. Generalizability is not possible, and furthermore, no causal relationships can be inferred from this study design. Furthermore, due to the focus on psychosocial factors, behavioral and biological factors, which are known to be important aspects in IBS, could only be considered marginally, e.g., diet, intolerances, and further comorbidities.

### Implications for research and practice

To deepen the understanding of social inequalities in IBS, more research is needed on individuals’ lived experiences of IBS symptoms and how and why these change over time, taking into account diverse social identities. LQR is well suited to reveal patterns of interactions and complex pathways but is not meant to show causation [52]. To enhance triangulation in future research [52], participants could be invited to reflect on the findings after a (certain) period of time, e.g. in focus group discussions. Here one could also make use of peer interaction. Moreover, exploring the perspective of relatives in addition to those directly affected, would provide valuable insights. The findings regarding shortage of or inappropriate (healthcare) services are consistent with previous reports [53] and should be discussed with medical providers and in politics to establish practical implementation strategies for existing guidelines as well as needed adaptations on structural level. Again, focus groups could be a valuable approach to gather all relevant stakeholders, including those affected by IBS.

Further implications for practice can be derived from the results to mitigate existing social inequalities in IBS. An overarching approach at the structural level is crucial to achieve long-term symptom improvement in as many cases as possible and to address the impact of lacking symptom improvement, particularly for individuals with a low occupational status. Concluding from the study results, structural changes regarding employment are needed. For example, enhanced compensation for lower occupational positions is necessary to facilitate part-time employment options [54]. Furthermore, preventive, organizational and management strategies at the workplace need to be developed and assessed for their effectiveness, e.g. flexible work arrangements or working remote, where possible [13, 22]. For individuals experiencing unemployment, institutionalized and individualized support for job search and, where necessary, retraining programs is essential. This would enable individuals whose ongoing severe symptoms, which require immediate toilet access, to transition to more adaptable office-based roles, ideally with remote work capabilities. Moreover, adaptations in the medical sector are necessary. For instance, clinical guidelines should be adhered to, ensuring timely yet not premature diagnostics for all and the targeted provision of existing treatment options [53]. Sensitized and trained general practitioners, acting as reliable gatekeepers, can effectively steer patients through the multi-sectoral healthcare system to reduce inappropriate and repeated medical procedures [55]. In parallel, establishing more multidisciplinary low-threshold medical centers specialized in gastrointestinal symptoms could be beneficial, particularly with regard to severe cases or rare comorbidities. This also includes a paradigm shift towards a biopsychosocial approach in health highlighting the relevance of psychosocial distress. A trusting doctor-patient relationship, sympathetic and transparent communication, including legitimate medical explanations and the feeling of worthiness, are vital to reduce stigmatization, particularly intersectional stigmatization [14, 56]. Furthermore, patients, especially those with a low occupational status, should be supported in developing their own strategies to understand and handle both their symptoms and personality traits. Psychoeducational interventions are particularly well-suited for this purpose, of which more low-threshold offers are needed already accompanying the diagnostic process, if possible [57, 58]. Moreover, empowerment of affected individuals, especially from low SES groups, can be enhanced through peer support as well as education via mass and social media [59, 60].

## Conclusions

Applying the LQR approach, results indicate social inequalities in IBS symptom development, since participants with a high occupational status as well as males experienced considerably large improvements in their symptoms in contrast to participants with a low occupational status or females, respectively. Although psychosocial stressors – which might explain at least some of the inequalities – were faced by all participants, people with a low occupational status were more limited in their social life and affected by existential worries due to their symptoms. In contrast, people with a high occupational status described several coping strategies including improving working conditions and understanding and managing symptoms as well as (anxious) personality traits. Furthermore, stigma in medical contexts was experienced irrespective of social identities, although participants with a low occupational status specifically reported psychologization and female participants (with a history of migration) seemed especially affected by negative experiences indicating intersectional stigma. Intertwined with a lack of social power, perceived stigma might have led to a diagnostic/treatment gap and ongoing uncertainty regarding possible explanations for the symptomatology. To strengthen individuals’ social power, especially the ones with intersecting social identities associated with oppression, like low SES, female gender, or a history of migration, and thereby mitigate social inequalities in IBS symptom development, several interventions are needed: work-related interventions (e.g. sufficiently high compensation and retraining programs), adaptations in medical care (such as equal access to timely diagnostics and transparent communication), and psychoeducational interventions (via peer support and media to enhance health literacy and coping strategies). 

## Supplementary Information

Supplementary Material 1.

## Data Availability

The datasets used and/or analyzed during the current study are available from the corresponding author on reasonable request.
